# Effects of cigarette smoking and secondhand smoke exposure on physical frailty development among community‐dwelling older adults in Japan: Evidence from a 10‐year population‐based cohort study

**DOI:** 10.1111/ggi.14708

**Published:** 2023-10-26

**Authors:** Wei‐Min Chu, Yukiko Nishita, Chikako Tange, Shu Zhang, Kanae Furuya, Hiroshi Shimokata, Meng‐Chih Lee, Hidenori Arai, Rei Otsuka

**Affiliations:** ^1^ Department of Family Medicine Taichung Veterans General Hospital Taichung Taiwan; ^2^ Center for Tobacco Treatment and Management Taichung Veterans General Hospital Taichung Taiwan; ^3^ School of Medicine National Yang Ming Chiao Tung University Taipei Taiwan; ^4^ Department of Post‐Baccalaureate Medicine College of Medicine, National Chung Hsing University Taichung Taiwan; ^5^ Department of Epidemiology of Aging National Center for Geriatrics and Gerontology Obu Japan; ^6^ Graduate School of Nutritional Sciences Nagoya University of Arts and Sciences AIchi Japan; ^7^ Department of Family Medicine Taichung Hospital, Ministry of Health and Welfare Taichung Taiwan; ^8^ Institute of Population Sciences, National Health Research Institutes Miaoli County Taiwan; ^9^ College of Management, Chaoyang University of Technology Taichung Taiwan; ^10^ Institute of Medicine, Chung Shan Medical University Taichung Taiwan; ^11^ National Center for Geriatrics and Gerontology Obu Japan

**Keywords:** cigarette smoking, frailty, longitudinal studies, older adults, secondhand smoking

## Abstract

**Aim:**

This study explored longitudinally the relationship between smoking and secondhand smoke and the incidence of physical frailty in community‐dwelling Japanese older people.

**Methods:**

Data collected from the National Institute for Longevity Sciences‐Longitudinal Study of Aging database from 2002 to 2012 (third to seventh wave) among older adults aged ≥65 years were analyzed. Participants with physical frailty at baseline, as determined by the Cardiovascular Health Study criteria, missing data or who failed to attend follow ups were excluded. Data on current cigarette smoking and secondhand smoke exposure were collected from the third wave results. The generalized estimating equation model was used to examine the longitudinal relationships between smoking, secondhand smoke and subsequent frailty.

**Results:**

The final analysis included 540 participants with a mean age of 71.4 years (standard deviation 4.6). The generalized estimating equation analysis showed that, compared with non‐smokers, smokers were at significant risk of physical frailty (odds ratio [OR] 2.39, 95% confidence interval [CI] 1.21–4.74) after adjustment for multiple covariates; especially men (OR 3.75, 95% CI 1.76–8.00) and older adults aged ≥75 years (OR 4.12, 95% CI 1.43–11.87). Participants exposed to both smoking and secondhand smoke had a higher risk of physical frailty (OR 3.47, 95% CI 1.56–7.73) than non‐smokers without secondhand smoke exposure. Smokers exposed to secondhand smoke were associated with more risk of physical frailty (OR 9.03, 95% CI 2.42–33.77) compared with smokers without secondhand smoke exposure.

**Conclusions:**

Smoking, especially when combined with secondhand smoke exposure, is associated with future physical frailty among older adults. **Geriatr Gerontol Int 2024; 24: 142–149**.

## Graphical abstract

This study explored longitudinally the relationship between smoking and secondhand smoke and the incidence of physical frailty in community‐dwelling Japanese older people. Cigarette smoking was associated with a higher incidence of physical frailty; and secondhand smoke exposure had a hugely additive effect on the incidence of physical frailty among smokers. It is important for healthcare professionals to identify older adults at risk of frailty, and to take action to reduce cigarette smoke exposure in advance.

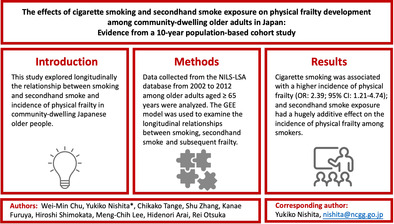

## Introduction

Smoking is the single biggest modifiable cause of morbidity and mortality in humans.^1^ According to the World Health Organization, in 2020, 22.3% of the global population used tobacco, representing 36.7% of all men and 7.8% of women globally.^2^ Tobacco smoking led to 7.69 million deaths and 200 million attributable disability‐adjusted life‐years (DALYs), accounting for 13.6% of all human deaths and 7.89% of all DALYs.^3^ Smoking is also a hazardous behavior among older adults specifically. It carries increased risk of dental caries, periodontitis, cancer, cognitive impairment, depression and mortality in older adults.^4^, ^5^, ^6^, ^7^

Secondhand and thirdhand smoking have also become a key focus of new research over the past decade. In 2017, 526 000 DALYs (0.36% of total DALYs) and 24 000 deaths (0.46% of total deaths) were attributable to home‐based secondhand smoke exposure in 28 EU countries, arising mainly from chronic obstructive pulmonary disease and ischemic heart disease.^8^ Furthermore, secondhand smoke increases the risk of cognitive impairment and tuberculosis infection.^9^, ^10^

Recently, healthy aging has become the central objective of care for older adults. An important aspect of healthy aging is prolonging life without disability or frailty. Frailty is associated with multimorbidity burden, poorer quality of life, future disability and even mortality.^11^, ^12^, ^13^ The mechanism underlying frailty consists of dysregulation of the innate body system with a decreased internal reserve for external stressful events, causing more damage among frail older adults. Risk factors of frailty include age, impaired cognitive function, poor self‐rated health, physical inactivity, malnutrition, polypharmacy and unemployment.^14^, ^15^, ^16^, ^17^, ^18^, ^19^

Previous studies have also reported a relationship between tobacco smoking and frailty. Kojima *et al*.^20^ discovered evidence of smoking as a predictor of worsening frailty status in a community‐dwelling population. Kaskirbayeva *et al*.^21^ identified smoking as one of the risk factors for the progression of frailty in older adults. Fu *et al*.^22^ used a registered database and found that not only does tobacco smoking negatively affect frailty prevention, but secondhand smoking also plays a role in determining frailty and pre‐frailty. Also, previous studies and meta‐analyses showed that there was a significantly increased likelihood of frailty in patients with chronic bronchitis or chronic obstructive pulmonary disease, and chronic obstructive pulmonary disease is a common chronic disease among people with a smoking habit. This evidence suggests that there is a possible link between smoking and frailty.^23^

However, most studies on secondhand smoke exposure and frailty are cross‐sectional, and the additive effect of secondhand smoke exposure on cigarette smoking is still unknown. Thus, this 10‐year longitudinal prospective cohort study explored the relationship between smoking and secondhand smoke and the incidence of physical frailty among community‐dwelling older adults in Japan.

## Methods

### Participants

Data were collected from the National Institute for Longevity Sciences, Longitudinal Study of Aging. The National Institute for Longevity Sciences, Longitudinal Study of Aging was a Japanese population‐based prospective cohort study of 2267 randomly selected men and women aged 40–79 years from Obu City and Higashiura Town in Aichi Prefecture, Japan. Seven waves of the study were carried out from 1997 to 2012. All participants provided written informed consent, and the Committee on Ethics of Human Research of the National Center for Geriatrics and Gerontology approved the study protocol. Details of the study have been described previously.^24^

**Figure 1:**
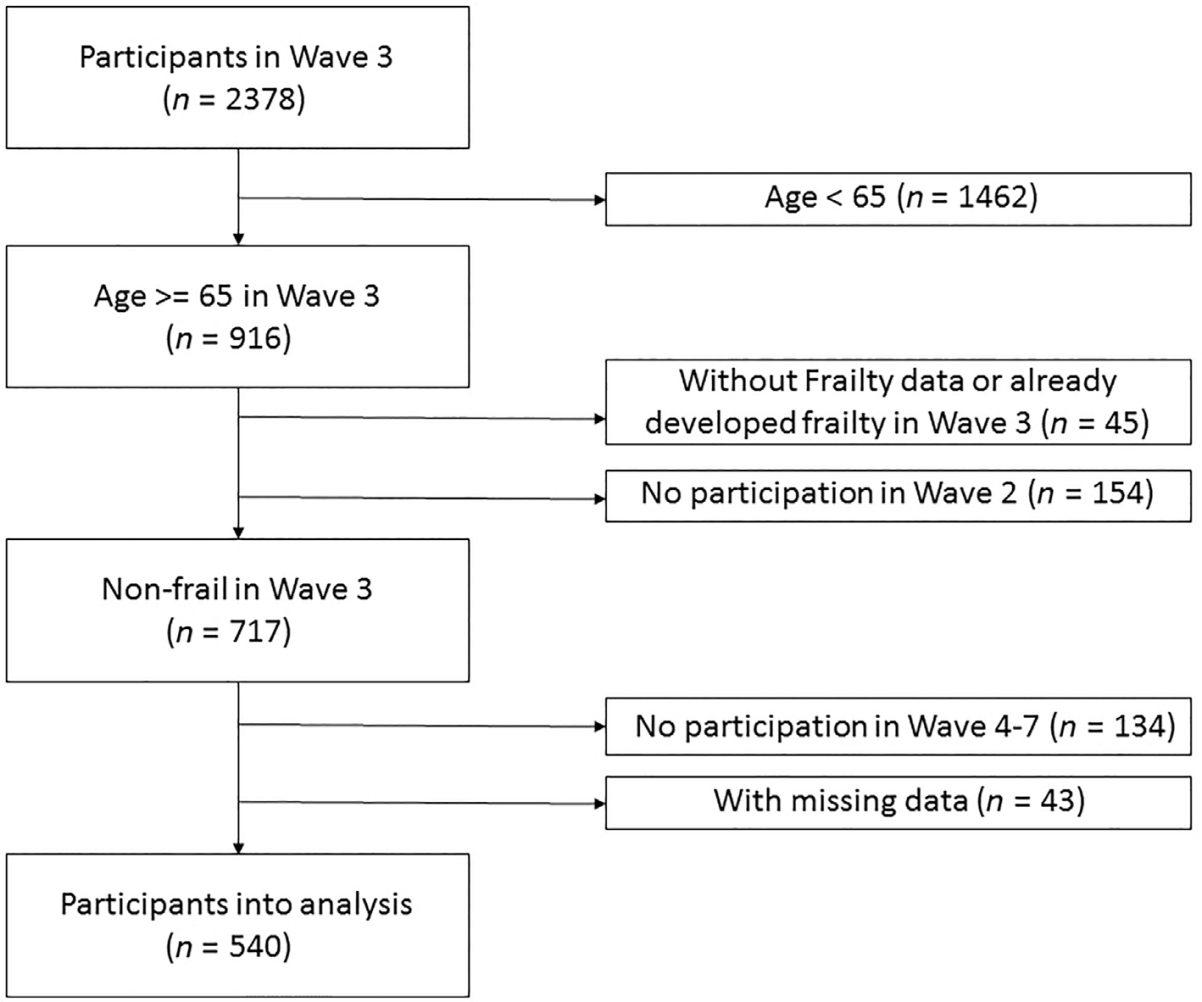
Flow chart of the study. GEE, generalized estimating equation; NILS‐LSA, National Institute for Longevity Sciences‐Longitudinal Study of Aging.

Data of the participants in this study were collected from the third wave in 2002. The third wave was chosen because it contained all variables used in this study. Initially, 2378 participants completed the measurements, with exclusions made under the following criteria: (i) aged <65 years (*n* = 1462); (ii) did not participate in the second wave, because weight loss was defined as a 5% weight loss in the past 2 years (*n* = 154); (iii) had missing physical frailty data during wave 3 (*n* = 15) or had already developed physical frailty in wave 3 (*n* = 30); (iv) could not attend any follow‐up investigations from the fourth to seventh waves (*n* = 134); and (v) had missing data on follow‐up investigation from the third to seventh waves (*n* = 43). Based on these inclusion and exclusion criteria, 540 participants were finally analyzed, with an average age of 71.4 years (standard deviation [SD] 4.6) and 6.6 years of follow up (Fig. 1).

### Measurements

#### Physical frailty

Physical frailty was determined using the modified cardiovascular health study criteria published in a previous study.^25^ Five domains of traits determined physical frailty: low grip strength, exhaustion, shrinking (weight loss), low gait speed and low activity.

Low grip strength was defined as a maximum grip strength <26 kg for men and <18 kg for women. A handgrip dynamometer (T.K.K.4301a; Takei Corporation, Niigata, Japan) was used to measure the grip strength of both hands during each wave of the study.

Exhaustion was measured by self‐report questionnaire, using Q7 “I could not get ‘going’” and Q20 “I felt that everything I did was an effort” of the Center for Epidemiologic Studies of Depression scale (CES‐D). Responses to these questions were: “Most or all of the time,” “Occasionally or a moderate amount of time,” “Some or a little of the time” and “Rarely or none of the time.” If the participants' response to these questions was not “Rarely or none of the time,” they were defined as having exhaustion.

Weight loss was defined as ≥5% weight loss within the preceding 2 years, based on weight change. Bodyweight was measured using two different digital scales: the first scale (T.K.K. 4342a; Takei Corporation) from the third to the fifth wave (until March 2008), and the second scale (WB‐510; TANITA Corporation, Tokyo, Japan) from the fifth to the seventh wave (from March 2008 to July 2012).^26^

Low gait speed was defined as a gait speed of <1.0 m/s, measured by a 10‐m walk test. Light sensors (YW‐3; Yagami, Aichi, Japan) were used during each wave of the study to determine the start and end of the walking test.

The level of leisure time activity was determined by interviews, based on the modified Minnesota Leisure‐Time Physical Activity Questionnaire, to assess leisure time activity (including activity intensity and frequency over the preceding year). Metabolic equivalents (METs) were assessed, and we calculated the mean values for physical activity (METs × min/year) during leisure time. Participants whose physical activity's lowest score was 20% of leisure‐time physical activity by sex were defined as having low physical activity.

Participants with deficits in zero to two domains were considered non‐frail, whereas participants with a deficit of three of more domains were considered frail.

#### Cigarette smoking and secondhand smoke exposure

At baseline, data on the conditions of cigarette smoking and secondhand smoke exposure were collected using a self‐administered questionnaire. All participants were asked: “Do you currently smoke cigarettes?” The answers were: “never,” “quit” or “currently smoking.” We regrouped the answers by combining non‐smokers and smokers who had quit into non‐smokers. For secondhand smoke exposure, each participant was asked two questions: “Does anyone in your family currently smoke?” and “Does anyone in the office, transportation or conference room currently smoke?” The answers were as follows: “no,” “sometimes” and “almost every day.” If the participants answered “no” to both questions, they were categorized into the group with no secondhand smoke exposure. If the participant answered “sometimes” or “almost every day” to either question, they were categorized into the group with secondhand smoke exposure.

#### Other variables

At baseline, the level of education (years), marital status (unmarried or married), depressive symptoms (CES‐D) and status of chronic diseases (hypertension, hyperlipidemia, heart disease, stroke and diabetes) were collected using a self‐administered questionnaire. Alcohol intake in the previous year was assessed using a food frequency questionnaire. The level of leisure time activity was determined by interviews, based on the modified Minnesota Leisure‐Time Physical Activity Questionnaire, to assess leisure time activity (including activity intensity and frequency over the preceding year). METs were assessed, and the mean values for physical activity (METs × min/year) during leisure time were calculated. Because oral health is strongly related to frailty, participants in wave 3 were examined by qualified dentists to determine the condition of their oral health and teeth. The judgment criteria included status and number of remaining teeth, type of restoration, condition of gingiva, with/without denture, habit of using denture, and amount of coating on the tongue.^27^, ^28^ Number of teeth was defined as the total number of remaining teeth during wave 3, and was categorized into two groups: ≥20 teeth and <20 teeth. The presence of serum minerals, such as magnesium, iron, lipid peroxide, calcium and phosphorus, were also measured, along with the participants' vitamin A and thyroid hormone levels.

### Statistical analysis

Sex distributions, marital status, smoking status, chronic disease status and number of teeth are shown as *n* (%). Age distribution, years of education, CES‐D and leisure‐time physical activity were reported as means and standard deviations. A generalized estimating equation (GEE) model was used to examine the longitudinal relationships between cigarette smoking, secondhand smoke exposure, and subsequent physical frailty in different models and adjusting covariates, including age, sex, years of education, marital status, employment status, leisure time activity, depressive symptoms, hypertension, stroke, heart disease, diabetes, hyperlipidemia, chronic bronchitis, number of teeth, lipid peroxide, magnesium, iron, calcium, phosphorus, free thyroxine (T4), thyroid‐stimulating hormone and vitamin A. Frailty was coded as a binary status in each wave, and the condition of cigarette smoking and secondhand smoke exposure at baseline was included into the GEE analysis separately to test the odds ratio (OR) and 95% confidence intervals (CI).

Statistical analyses were carried out using the sas System version 9.3 (SAS Institute, Cary, NC, USA). The GEE models were fitted using the GENMOD procedure in sas. A two‐tailed *P*‐value <0.05 was considered statistically significant.

## Results

Table 1 shows follow‐up participation details. The mean follow‐up duration from baseline to final assessment was 6.6 years. A total of 1693 cumulative observations from 540 participants were analyzed in the present study.

**Table 1:** Information on follow‐up participation

	Total, *n* (%)	Follow‐up years from baseline, mean (SD)	Physical frailty, *n* (%)^†^
Wave 3 (baseline)	540 (100.0)	0.00	–
Wave 4	530 (98.2)	2.14 (0.10)	32 (6.2)
Wave 5	453 (83.9)	4.20 (0.17)	38 (8.7)
Wave 6	387 (71.7)	6.19 (0.17)	39 (10.6)
Wave 7	323 (59.8)	8.16 (0.25)	30 (9.7)
Cumulative data	1693		139

^†^ The percentage was calculated as (*n* of physical frailty at each study wave) / (total *n* at each study wave) × 100.

Eventually, 540 participants with an average age of 71.4 years (SD 4.6) were enrolled. Table 2 shows the basic characteristics of participants; 52.4% were male; they were mostly non‐smokers (smokers 13.2%), married (79.6%), non‐employed (72.8%) and with ≥20 teeth (54.8%). Average educational years were 10.9, and baseline CES‐D was 7.0. The participants had an average of 48 477.4 METS × min/year.

**Table 2:** Baseline characteristics of all participants.

	All participants (*n* = 540)
Variables	*n* (%)
Sex	
Male	283 (52.4)
Female	257 (47.6)
Marital status	
Married	430 (79.6)
Not married	110 (20.4)
Hypertension	211 (39.1)
Stroke	26 (4.8)
Heart disease	53 (9.8)
Diabetes	49 (9.1)
Hyperlipidemia	130 (24.1)
Chronic bronchitis	19 (3.5)
Employed	147 (27.2)
Smoking	71 (13.2)
No. teeth	
≥20	296 (54.8)
<20	244 (45.2)

	Mean (SD)
Age (years)	71.4 (4.6)
Years of education	10.9 (2.5)
Alcohol intake (g/day)	7.9 (15.4)
Leisure time activity (METs × min/year)	48 477.4 (55 176.1)
Lipid peroxide (nmoL/mL)	3.0 (0.9)
Magnesium (mg/dL)	2.4 (0.2)
Iron (μg/dL)	109.8 (35.4)
Calcium (mg/dL)	9.4 (0.3)
Phosphorus (mg/dL)	3.4 (0.4)
Free T4 (ng/dL)	1.2 (0.2)
TSH (μIU/mL)	3.4 (5.5)
Vitamin A (IU/dL)	194.8 (47.4)
CES‐D	7.0 (6.7)

Abbreviations: CES‐D, Center for Epidemiologic Studies of Depression scale; METs, metabolic equivalents; SD, standard deviation; T4, thyroxine; TSH, thyroid‐stimulating hormone.

Table 3 shows the differences between smokers (*n* = 71) and non‐smokers (*n* = 469) at baseline. The distribution of age, sex, marital status, employment status, alcohol consumption, CES‐D and chronic diseases, such as hyperlipidemia, was significantly different, whereas the distribution of years of education, leisure time activity and chronic diseases, such as hypertension, stroke, chronic bronchitis and diabetes, did not differ significantly. For serum minerals, vitamin A and thyroid hormones, the distribution of phosphorus and free T4 differed significantly between smokers and non‐smokers. Compared with non‐smokers, smokers had higher levels of free T4 and lower phosphorus levels.

**Table 3:** Demographics and characteristics of participants with/without smoking in wave 3

Variables	Smoking status		*P*‐value
	Without smoking (*n* = 469)	With smoking (*n* = 71)	
	*n* (%)	*n* (%)	
Sex			<0.0001
Male	217 (46.3)	66 (93.0)	
Female	252 (53.7)	5 (7.0)	
Marital status			0.0183
Married	366 (78.0)	64 (90.1)	
Not married	103 (22.0)	7 (9.9)	
Hypertension	187 (39.9)	24 (33.8)	0.3296
Stroke	20 (4.3)	6 (8.5)	0.1251
Heart disease	42 (9.0)	11 (15.5)	0.0847
Diabetes	41 (8.7)	8 (11.3)	0.4908
Hyperlipidemia	123 (26.2)	7 (9.9)	0.0026
Chronic bronchitis	14 (2.99)	5 (7.04)	0.0841
Employed	115 (24.5)	32 (45.1)	0.0003

	Mean (SD)	Mean (SD)	
Age (years)	71.6 (4.6)	70.1 (4.4)	0.0082
Years of education	10.9 (2.5)	11.2 (2.4)	0.2689
Alcohol intake (g/day)	6.7 (12.0)	16.1 (28.2)	<0.0001
Leisure time activity (METs × min/year)	49 790.7 (54 332.9)	39 801.8 (60 144.5)	0.1553
Lipid peroxide (nmoL/mL)	3.0 (0.9)	3.1 (0.8)	0.4956
Magnesium (mg/dL)	2.4 (0.2)	2.3 (0.2)	0.3697
Iron (μg/dL)	108.8 (35.3)	115.8 (35.9)	0.1226
Calcium (mg/dL)	9.4 (0.3)	9.3 (0.3)	0.1874
Phosphorus (mg/dL)	3.5 (0.4)	3.3 (0.4)	0.0067
Free T4 (ng/dL)	1.2 (0.2)	1.3 (0.2)	0.0272
TSH (μIU/mL)	3.4 (5.4)	3.1 (5.7)	0.6406
Vitamin A (IU/dL)	194.6 (47.2)	196.5 (5.7)	0.7525
CES‐D	6.7 (6.7)	8.4 (6.7)	0.0467

Abbreviations: CES‐D, Center for Epidemiologic Studies of Depression scale; METs, metabolic equivalents; SD, standard deviation; T4, thyroxine; TSH, thyroid‐stimulating hormone.

Table 4 shows the GEE analysis for the relationship between cigarette smoking and the risk of physical frailty with multivariate analysis in different models. After adjusting for age, sex, marital status, years of education, employment status, leisure time activity, depressive symptoms, hypertension, stroke, heart disease, diabetes, hyperlipidemia, chronic bronchitis, number of teeth, lipid peroxide, magnesium, iron, calcium, phosphorus, free T4, thyroid‐stimulating hormone and vitamin A, smoking was significantly (*P* < 0.05) associated with a higher risk of physical frailty (OR 2.39, 95% CI 1.21–4.74). Considering secondhand smoke exposure, both cigarette smoking and secondhand smoke exposure were also significantly (*P* < 0.05) associated with a higher risk of physical frailty (OR 3.47, 95% CI 1.56–7.73) compared with non‐smokers without secondhand smoke exposure. However, non‐smokers with secondhand smoke exposure, or smokers without secondhand smoke exposure had no significant relationship with the risk of physical frailty.

**Table 4:** Association of cigarette smoking, secondhand smoke exposure and incident frailty by multivariate generalized estimating equation analysis

	Model 1^†^		Model 2^‡^		Model 3^§^		Model 4^¶^	
Variable	OR	95% CI	OR	95% CI	OR	95% CI	OR	95% CI
Smoking status								
No smoking	Ref		Ref		Ref		Ref	
Smoking	2.43	1.30–4.54	2.67	1.39–5.13	2.35	1.16–4.76	2.39	1.21–4.74
Smoking status and secondhand smoke exposure								
No smoking, no secondhand smoke	Ref		Ref		Ref		Ref	
No smoking, with secondhand smoke	1.19	0.76–1.85	1.01	0.64–1.59	1.11	0.70–1.74	1.15	0.73–1.83
With smoking, no secondhand smoke	1.36	0.51–3.66	1.43	0.54–3.84	1.2	0.39–3.67	1.17	0.41–3.39
With smoking, with secondhand smoke	3.63	1.73–7.61	3.53	1.58–7.86	3.25	1.43–7.41	3.47	1.56–7.73

^†^ Adjusted with age and sex.

^‡^ Adjusted with age, sex, years of education, marital status, employment status, leisure time activity and depressive symptoms.

^§^ Adjusted with age, sex, years of education, marital status, employment status, leisure time activity, depressive symptoms, hypertension, stroke, heart disease, diabetes, hyperlipidemia and number of teeth.

^¶^ Adjusted with age, sex, years of education, marital status, employment status, leisure time activity, depressive symptoms, hypertension, stroke, heart disease, diabetes, hyperlipidemia, chronic bronchitis, number of teeth, lipid peroxide, magnesium, iron, calcium, phosphorus, free thyroxine, thyroid‐stimulating hormone and vitamin A.

Abbreviations: CI, confidence interval; OR, odds ratio.

Table 5 shows subgroup analysis of the association between cigarette smoking and the incidence of physical frailty among participants of different sex and age. The effect of cigarette smoking on the incidence of physical frailty only showed among male participants, with OR 3.75 and 95% CI 1.76–8.00 in multivariate analysis. For analysis among different age groups, participants aged ≥75 years had more risk of developing physical frailty if they were cigarette smokers (OR 4.12, 95% CI 1.43–11.87).

**Table 5:** Subgroup analysis of association with cigarette smoking and physical frailty among participants of different sex and age by multivariate generalized estimating equation analysis

	Male								Female							
	Model 1^†^		Model 2^‡^		Model 3^§^		Model 4^¶^		Model 1^†^	Model 2^‡^			Model 3^§^		Model 4^¶^	
Variable	OR	95% CI	OR	95% CI	OR	95% CI	OR	95% CI	OR	95% CI	OR	95% CI	OR	95% CI	OR	95% CI
Smoking status																
No smoking	Ref		Ref		Ref		Ref		Ref		Ref		Ref		Ref	
Smoking	3.01	1.51–6.00	3.7	1.88–7.31	3.25	1.54–6.89	3.75	1.76–8.00	0.63	0.08–4.88	0.46	0.05–4.34	0.35	0.03–4.36	0.34	0.02–4.85

^†^ Adjusted with age.

^‡^ Adjusted with age, years of education, marital status, employment status, leisure time activity and depressive symptoms.

^§^ Adjusted with age, years of education, marital status, employment status, leisure time activity, depressive symptoms, hypertension, stroke, heart disease, diabetes, hyperlipidemia and number of teeth.

^¶^ Adjusted with age, years of education, marital status, employment status, leisure time activity, depressive symptoms, hypertension, stroke, heart disease, diabetes, hyperlipidemia, chronic bronchitis, number of teeth, lipid peroxide, magnesium, iron, calcium, phosphorus, free thyroxine, thyroid‐stimulating hormone and vitamin A.

**Table:** 

	Age <75 years								Age ≥75 years							
Smoking status																
No smoking	Ref		Ref		Ref		Ref		Ref		Ref		Ref		Ref	
Smoking	2	0.83–4.82	1.98	0.84–4.67	1.83	0.76–4.68	2.18	0.86–5.53	3.06	1.21–7.76	4.28	1.63–11.25	3.7	1.14–12.00	4.12	1.43–11.87

^†^ Adjusted with age and sex.

^‡^ Adjusted with age, sex, years of education, marital status, employment status, leisure time activity, and depressive symptoms.

^§^ Adjusted with age, sex, years of education, marital status, employment status, leisure time activity, depressive symptoms, hypertension, stroke, heart disease, diabetes, hyperlipidemia and number of teeth.

^¶^ Adjusted with age, sex, years of education, marital status, employment status, leisure time activity, depressive symptoms, hypertension, stroke, heart disease, diabetes, hyperlipidemia, chronic bronchitis, number of teeth, lipid peroxide, magnesium, iron, calcium, phosphorus, free thyroxine, thyroid‐stimulating hormone and vitamin A.

Abbreviations: CI, confidence interval; OR, odds ratio.

Table 6 shows the negative effect of secondhand smoke exposure among cigarette smokers. Compared with smokers without secondhand smoke exposure, smokers with secondhand smoke exposure had a higher risk of physical frailty, with OR 9.03 and 95% CI 2.42–33.77 in multivariate analysis.

**Table 6:** Association of secondhand smoke exposure and incident frailty among cigarette smokers by multivariate generalized estimating equation analysis

	Model 1^†^		Model 2^‡^		Model 3^§^	
Variable	OR	95% CI	OR	95% CI	OR	95% CI
Secondhand smoke exposure						
No	Ref		Ref		Ref	
Yes	2.46	0.92–6.58	4.82	1.16–20.11	9.03	2.42–33.77

^†^ Adjusted with age and sex.

^‡^ Adjusted with age, sex, years of education, marital status, employment status, leisure time activity and depressive symptoms.

^§^ Adjusted with age, sex, years of education, marital status, employment status, leisure time activity, depressive symptoms, hypertension, stroke, heart disease, diabetes, hyperlipidemia and number of teeth.

Abbreviations: CI, confidence interval; OR, odds ratio.

## Discussion

To the best of our knowledge, this is the first study to longitudinally explore the relationship between cigarette smoking, secondhand smoke exposure and physical frailty in Japanese older adults. We discovered that cigarette smoking was associated with a higher incidence of physical frailty; and secondhand smoke exposure had a hugely additive effect on the incidence of physical frailty among smokers.

These results are consistent with those of previous studies. In Korea, Shin *et al*. investigated 1426 community‐dwelling older adults aged >70 years, and discovered that frailty in the smoking and non‐alcohol‐intake groups was significantly higher according to the cardiovascular health study frailty index (OR 1.59, 95% CI 1.03–2.46).^29^ In a UK‐based study, Kojima *et al*. found that current smokers were twice as likely to develop frailty compared with non‐smokers (OR 2.07, 95% CI 1.39–3.39), with this association largely attenuated by controlling for socioeconomic status.^30^ In the USA, Crane *et al*.^31^ carried out a study among people with HIV, and found that current smoking was associated with a 61% higher risk of being frail versus not frail (OR 1.61, 95% CI 1.41–1.85) in adjusted analyses. The results of the present study strengthen the association between tobacco smoking and frailty in the older Japanese population.

Although there have only been a few cross‐sectional studies on the relationship between secondhand smoke exposure and physical frailty, the present results also showed that secondhand smoke has an additive effect on physical frailty. Akhtar^32^ found that secondhand smoke is associated with decreased gait speed in both non‐smokers and former smokers, based on the US National Health and Nutrition Examination Survey data. Secondhand smoke is also associated with a higher incidence of both pre‐frailty and frailty. Fu *et al*.^22^ investigated the data of non‐smokers obtained from the US National Health and Nutrition Examination Survey, and found that higher serum cotinine levels are associated with an increased risk of pre‐frailty and frailty in non‐smoking older adults. Another study that used US National Health and Nutrition Examination Survey data observed an increased frequency of frailty in participants who reported living with two or more smokers at home (OR 5.37, 95% CI 1.13–25.5).^33^ However, these were all cross‐sectional studies. The present study is the first to longitudinally investigate the additive effect of secondhand smoke on the incidence of frailty among smokers.

The relationship between active cigarette smoking and secondhand smoking is also important. Lee *et al*.^34^ carried out a study in African countries, and found that the majority of never smokers who were exposed to secondhand smoke at home and in public places had a higher prevalence of susceptibility to initiate smoking than those that were not exposed to secondhand smoke at home and in public places. This shows that the coexistence of secondhand smoke exposure and active smoking is common. From the present results, participants with both active smoking and secondhand smoke exposure had a significantly higher chance to develop physical frailty, whereas secondhand smoke exposure had a hugely additive effect on the incidence of physical frailty among smokers. We believe that the present results provide valuable information on the combined effect of active smoking and secondhand smoke exposure to the development of frailty, whereas healthcare professionals and policy makers should care more about the surrounding environment of older smokers who has a long smoking history.

Chronic inflammation is a popular explanation for the link between smoking and frailty.^20^ Elevated levels of inflammatory markers, such as interleukin‐6 and C‐reactive protein, have been found among both frail older adults and smokers.^35^, ^36^ There also exists an association between smoking and lipid peroxidation, with lipid peroxidation playing a role in inflammatory processes and contributing to oxidative stress.^37^, ^38^ However, the present results show that the distribution of lipid peroxides was not significantly different between smokers and non‐smokers. Instead, there were some differences in phosphorus and free T4 levels between smokers and non‐smokers, but no clear evidence of an association between phosphorus or hyperthyroidism and frailty. Recently, researchers found that epigenetic alterations with deoxyribonucleic acid methylation play a role in the smoking‐associated development of frailty. Furthermore, secondhand smoke exposure is also associated with deoxyribonucleic acid methylation.^39^, ^40^ The present findings show that secondhand smoke is also important in the development of frailty, and the role of deoxyribonucleic acid methylation should be explored further in future studies.

The present study had several strengths. First, the National Institute for Longevity Sciences, Longitudinal Study of Aging is a regional population‐based cohort study; therefore, the sample was representative of the general population in Japan. Second, the participants were followed up for an average of 7.3 years with detailed measurements taken each time. Third, this is the first study to longitudinally explore the relationship between cigarette smoking, secondhand smoke exposure and physical frailty in Japanese older adults.

However, this study also had limitations. First, only healthy Japanese older adults were enrolled who could independently travel to the survey center to undergo investigations; thus, the external validity needs to be carefully interpreted. Second, some objective measurements that could be related to physical frailty, such as body composition or imaging, were not included in the study. However, some important objective tests were carried out, such as height, weight, grip strength, walking speed and physical activity, using the METs, to compensate for the possible bias of residual confounders. Third, chronic inflammation might lead to frailty, and the level of inflammation is related to cumulative dose of smoking; however, the cumulative pack years of smoking dose was not ascertained in the present study. Future research is warranted to explore the if there is a dose–response relationship between cigarette smoking and incidence of frailty among older adults. Fourth, there is no validation reference for measurement regarding secondhand smoke exposure, so the validity and reliability of the questions need to be tested in the future.

The present study highlights the importance of cigarette smoking and secondhand smoke exposure, and their relationship with the development of frailty. Cigarette smoking and secondhand smoke exposure could be prevented by increasing tobacco cessation programs, public education and public health measures. It is important for healthcare professionals to identify older adults at risk of frailty, and to take action to reduce cigarette smoke exposure in advance.

## Disclosure statement

The authors declare no conflict of interest.

## Funding information

This work was supported by Research Funding for Longevity Sciences from the National Center for Geriatrics and Gerontology, Japan (grant number 20‐1; 21‐18).

## Ethics statement

This study was approved by the Institutional Review Board of the National Center for Geriatrics and Gerontology, Japan (Case number: 899‐6).

## Data Availability

The data that support the findings of this study are available from the corresponding author upon reasonable request.
